# Prediction of the Compressive Strength of Fly Ash Geopolymer Concrete by an Optimised Neural Network Model

**DOI:** 10.3390/polym14071423

**Published:** 2022-03-31

**Authors:** Ali Abdulhasan Khalaf, Katalin Kopecskó, Ildiko Merta

**Affiliations:** 1 Department of Engineering Geology and Geotechnics, Faculty of Civil Engineering, Budapest University of Technology and Economics, 1111 Budapest, Hungary; ali.abdulhasan.khalaf@emk.bme.hu (A.A.K.); kopecsko.katalin@emk.bme.hu (K.K.); 2 Building Physics and Building Ecology, Institute of Material Technology, Faculty of Civil Engineering, TU Wien, 1040 Vienna, Austria

**Keywords:** fly ash, geopolymer concrete, alkali-activated binder, compressive strength, feedforward layered neural network, Matlab code, optimisation, design chart

## Abstract

This article presents a regression tool for predicting the compressive strength of fly ash (FA) geopolymer concrete based on a process of optimising the Matlab code of a feedforward layered neural network (FLNN). From the literature, 189 samples of different FA geopolymer concrete mix-designs were collected and analysed according to ten input variables (all relevant mix-design parameters) and the output variable (cylindrical compressive strength). The developed optimal FLNN model proved to be a powerful tool for predicting the compressive strength of FA geopolymer concrete with a small range of mean squared error (MSE = 10.4 and 15.0), a high correlation coefficient with the actual values (R = 96.0 and 97.5) and a relatively small root mean squared error (RMSE = 3.22 and 3.87 MPa) for the training and testing data, respectively. Based on the optimised model, a powerful design chart for determining the mix-design parameters of FA geopolymer concretes was generated. It is applicable for both one- and two-part geopolymer concretes, as it takes a wide range of mix-design parameters into account. The design chart (with its relatively small error) will ensure cost- and time-efficient geopolymer production in future applications.

## 1. Introduction

The high CO_2_ gas emissions in the process of cement production have raised the need to seek new alternatives to Portland cement because the latter is well known for its high carbon footprint. Manufacturing approximately 1 ton of cement produces 1 ton of CO_2_ gas due to the energy consumption and decarbonisation process of limestone [1].

Promising alternatives for replacement cement are novel sustainable binders, namely the so-called geopolymer (or alkali-activated) binders consisting of amorphous silico-aluminate phases such as metakaolin, ground granulated blast furnace slag (GGBFS), silica fume or fly ash (FA) [2,3,4]. Geopolymers are characterised as having a considerably low carbon footprint and potential as a future green building material [5]. Geopolymers exhibit binding properties in the presence of an alkaline environment such as sodium hydroxide, sodium silicate, or a mix of them to produce three-dimensional structures of silico-aluminate consisting of sialate (Si-O-Al) and siloxo (Si-O-Si) bonds at low temperatures [2,3,4,6]. Geopolymers can be synthesised either by mixing the raw material with an alkali activator in a liquid state (the so-called two-part geopolymers), where one part of the reacted material (the raw material) is in a separate phase from the other reacted material (the alkali activator), or in a solid state (the so-called one-part geopolymers) by just adding water because both reacted materials are in the same solid phase [4].

Generally, in the mix-design of geopolymer concrete, the same method is utilised as in traditional Portland cement concretes, taking the compressive strength of the materials as the cardinal design characteristic. The compressive strength (f’c) of concrete is a function of its mix-design proportion and of the water–cement (w/c) ratio of the matrix, and thus it can be predicted according to the proportion of the particular ingredients [7]. Most design codes, such as ACI 318 [8] and the CEB FIP model [9], express the other concrete properties, such as its tensile strength, modulus of elasticity and flexural strength, as a function of its compressive strength [7,8,9]. Thus, by knowing the material’s compressive strength, the engineers are able to determine all the other necessary material properties for preliminary design purposes.

Lloyd and Rangan [10] proposed a mix-design method assuming that the geopolymer concrete’s density equals the density of the Portland cement concrete. Pavithra et al. [11] proposed a mix-design method based on Indian standards for Portland cement concrete. These approaches showed satisfying results as a starting point for defining the geopolymer concrete mix-design; however, they were based on fixing the alkali solution’s composition which did not provide sufficient flexibility in the design of the matrix.

Statistical analysis is another approach to geopolymer concrete mix-design that is based on determining the relationship between the composite’s mix ingredients and its compressive strength. These methods are the Taguchi method [12,13], linear multivariate regression [12,14] and nonlinear multivariate regression [12,14]. In the study conducted by Ahmed [14], the performance of the linear multivariate regression and nonlinear multivariate regression models were compared with the same input variables. The nonlinear model showed lower RMSE values than the linear model for the training and validation subsets. It was found that the linear relationship between the geopolymer concrete’s ingredients and its compressive strength was not adequate, but the relationship is expected to be more nonlinear [12]. In statistical analysis approaches, the fixed composition of the alkali solution again limits the flexibility of the design [15].

Machine learning techniques are powerful tools for solving statistical problems in engineering challenges. The most prominent techniques are artificial neural networks (ANNs) (commonly called neural networks (NNs)), multivariate regression (MR) and gene expression programming (GEP). So far, they have been successfully utilised in different areas of civil engineering and building materials science [14,16,17,18]. Some of the most widely used techniques are the NNs, which serve as a typical tool for regression problems and are able to capture the complex relationships of material input variables [16,19].

The single-layer perceptron type of NN is only suitable for linear relationships, while a multilayer perceptron allows more complex computations such as nonlinearity. The choice of NN depends on the learning task [19,20]. Since the compressive strength of geopolymer concrete is considered to be a nonlinear function [12,14], the multilayer perceptron type would overcome the limitations of a single-layer NN and of the least mean squared algorithm. The reason why a multilayer perceptron can overcome the limitations of the single-layer NN is because of the behaviour of the hidden layer, the activation function and the high degree of connectivity, which can handle nonlinearity [20].

In regression problems, a feedforward neural network (FNN) is more suitable than other types of machine learning (MR and GEP) because it is powerful in computation. It is a very strong and robust tool in the analysis of error signals [16,18,20]. One powerful learning method is when an NN is provided with a randomly picked example from the set, modifying the synaptic weights to minimise the error between the model response and the experimental response of the network based on the statistical procedure [20]. The learning is characterised as supervised learning or learning with a teacher by using a set of labelled training examples. This type of learning can represent the knowledge of the environment in the network. Thus, the best choice [19,20] of NN type for nonlinear regression problems is a feedforward layered neural network (FLNN) with supervised learning [16,20].

Hardjito and Rangan [21] and Lloyd and Rangan [10] have conducted extensive experimental work on FA geopolymer concrete. The most important conclusion of their work was introducing the water–geopolymer solid (w/GS) ratio. When this term increases, the compressive strength decreases. They also concluded that the compressive strength increases with an increase in the curing temperature and concentration of sodium hydroxide. As a result, Lloyd and Rangan [10] introduced their mix-design method for FA geopolymer concrete based on a fixed ratio of an alkali solution (0.30–0.45) with a narrow range of w/GS (i.e., 0.17–0.22) and only four temperature levels (30 °C, 45 °C, 75 °C and 90 °C). However, the chemical composition of FA has not been taken into consideration and it is only applicable for two-part geopolymers. Diaz [22] studied the linear MR of 32 mixes that followed the mix-design method in [10,21], considering the chemical composition of FA. The results yielded MSE and R values of 75.18% and 84.9%, respectively. Toufigh and Jafari [23] collected 162 different mixes of FA geopolymer concrete to develop an MR model that predicted the compressive strength by considering 12 input variables including five major oxides of the FA’s chemical composition. They introduced a linear model with 25 model parameters, which required tedious calculation and a long time to determine a specific mix. The RMSE, MSE and R values were 4.83 MPa and 5.96 MPa, 23.3 MPa^2^ and 35.5 MPa^2^ and 89.0% and 82.6% for the training and testing subsets, respectively. The statistical parameters of RMSE and R did not achieve good values and could be improved, and the model is only applicable for two-part geopolymers. Furthermore, their model was built by considering a linear relationship, which is not the best representation, as has been proven by Ahmed et al. [14]. Ahmed et al. [14] undertook a comparison study of compressive strength models of FA geopolymer concrete using MR. Their conclusion was that models that considered the nonlinear relationship produced the best results, with R values of 93.0% and 87.0% for the training and testing subsets, respectively, while the RMSE value was 4.19 MPa for the training subset (the RMSE value was not reported for the testing subset). This model may be overfitted because there was a considerable difference between the R values of the training and testing subsets (0.06%) and the RMSE value for the testing subset did not provide complete information about the average deviation (in units of MPa) of the predicted value from the actual value for future applications. Moreover, this model did not consider all five major oxides of FA chemical composition but only considered SiO_2_ and Al_2_O_3_, and it is only applicable for two-part geopolymers.

So far, no comprehensive NN model has been published in the literature for predicting the compressive strength of FA-based geopolymer concretes that is applicable to both one- and two-part geopolymer concretes and that simultaneously considers the wide range of the composite’s possible design parameters.

This research aimed to build a comprehensive design model by optimising a code based on the FLNN as a regression tool to predict the compressive strength of FA-based geopolymer concrete using Matlab R2020b software provided by The MathWorks, Inc.; 1 Apple Hill Drive Natick, MA, USA under the license’s number ‘40552969’ [24]. The significance of the model developed here is that it takes the wide range of possible mix-design (input) parameters into account; moreover, because of its reduced error signal, it has high reliability in predicting the compressive strength of FA-geopolymer concretes.

Based on the optimal FLNN model, a powerful practical design chart for determining the mix-design parameters of both one- and two-part geopolymer concretes has been generated by taking the wide range of possible mix-design parameters into account. The design chart will enable cost- and time-efficient production of geopolymers, thus supporting engineers in broader applications of this novel material in the future practice.

**Figure 1:**
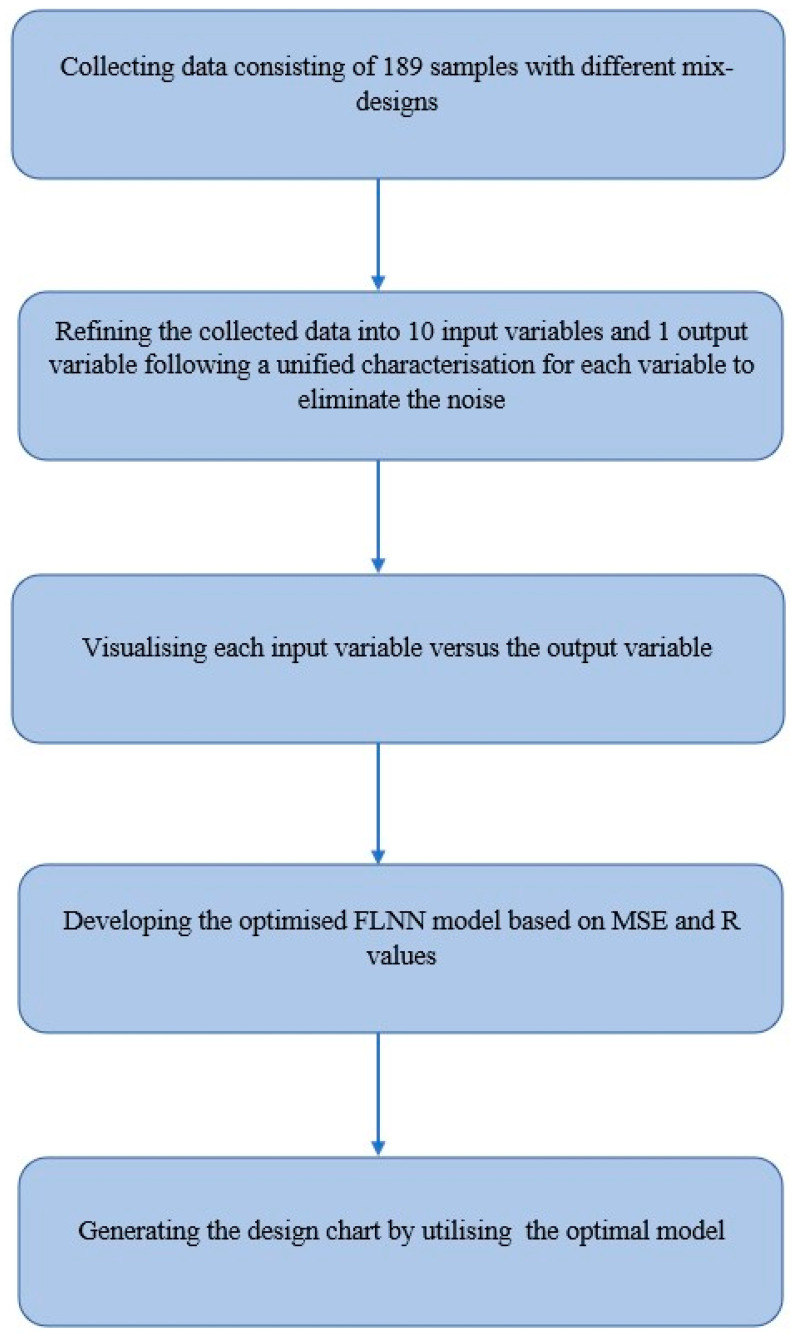
Graphical outline of the study design.

A graphical outline of the study design is depicted in Figure 1.

## 2. Materials and Methods

In Section 2.1, the methodology of the FLNN as a regression tool is discussed, starting from the theoretical basis of using FLNN for function approximation and formulating the regression equation. Section 2.2 discusses the functional form of the compressive strength of FA-based geopolymer concrete and its connection with the FLNN regression equation by formulating the objective function to produce the Hessian matrix. The section then describes the evaluation performance criteria of the FLNN model and the method of testing its reliability. Section 2.3 describes the materials used in developing the Matlab code. For the materials in the dataset, we describe how they were collected and analysed based on the previous findings so that the analysis would be effective.

### 2.1. FLNN for Function Approximation

**Figure 2:**
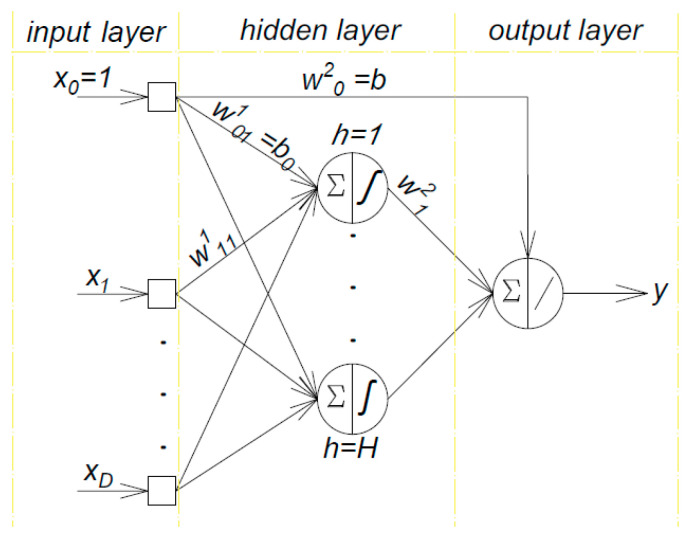
The architecture of the FLNN with one hidden layer.

According to the universal approximation theorem [20], one hidden layer is enough to represent a multilayer perceptron in the function approximation process based on error correction learning. The functional form of the FLNN with one hidden layer was obtained by the following equation [19,20]:

(1) $$y\left(x;w\right)=\overset{H}{\underset{h=1}{\sum }}w_{h}^{\left(2\right)}\;*\;g\;*\;\left(\overset{D}{\underset{j=1}{\sum }}w_{\mathrm{hj}}^{\left(1\right)}{\;*\;x}_{j}+w_{0j}^{\left(1\right)}\right)+w_{0}^{\left(2\right)}$$

where y (**x**; **w**) is the model response, **x** is the input vector, **w** is the vector of the free parameters (weights and biases), H is the number of neurons in the hidden layer, g is the activation function in the hidden layer, D is the input number, 1 and 2 are the numbers of layers, h = 1 − H, j = 1 − D and w_0j_ and w_0_ are the bias parameters for the first and second layers, respectively. The architecture of this kind of neural network is depicted in Figure 2.

### 2.2. Definition of the Compressive Strength Function FA-Based Geopolymer Concretes

The compressive strength of geopolymer concrete (output variable) is a nonlinear function of the proportion of the ingredients (input variables). The following equation describes the functional relationship between the input and output variables [20]:

(2) d=f(x),

where **x** is the input vector, **d** is the output vector and **f**(**x**) is an unknown vector-valued function. However, the training set (T) is responsible for providing the required knowledge about the **f**(**x**) function and is described as follows:

(3) $$T={\left\{\left(x_{i},d_{i}\right)\right\}}_{i=1}^{N},$$

where N is the size of the training set.

The goal of the neural network is to construct an approximation function (**F**(**x**)) which is close enough to the unknown function (**f**(**x**)) by utilising the input–output mapping as follows:

(4) ‖F(x)−f(x)‖< ε for all x,

where ε should be a small positive number and the minimisation (of the error) is dependent on the size of the training set (N) and of the free parameters (**w**) of the structure of the FLNN. Therefore, the function approximation is expressed as follows:

(5) F(x)=y(x,w),

#### 2.2.1. The Objective Function and Operation Mechanism of the FLNN

By training the FLNN to produce the system model, the difference between the FLNN output vector (**y_i_**) and the experimental output vector (**d_i_**) provides the error signal vector (**e_i_**) [20]. When the model overestimates or underestimates the actual response, the error signal will be either a positive or a negative value, respectively. As a result, choosing the squared error prevents some polynomials being diminished when calculating the mean value [25]. The optimisation problem is to reduce the mean squared error. The free parameters are adjusted through the training process by various learning algorithms. The objective function is written as follows [20,26]:

(6) $$E\left(w\right)=\frac{1}{N}*\sum_{i=1}^{N}{\left(e_{i}\right)}^{2},$$

The only unknown is **w** (the vector of the free parameters). Thus, the objective function is a function of **w**. Since the goal is to minimise the objective function, the optimisation problem will be unconstrained. The mean squared function represents the error surface, and the free parameters are the coordinates in the learning process. The most important criteria for all learning algorithms are the objective function gradient and the Hessian matrix (**H**). The gradient is the first partial derivative of the objective function. Still, the Hessian matrix is the second partial derivative of the objective function with respect to the free parameters **w**, as stated in Equation (7) [20]:

(7) $$H=\nabla^{2}E\left(w\right)=\left[\begin{array}{ccc} \begin{array}{cc} \frac{\partial^{2}E}{\partial w_{1}^{2}} & \frac{\partial^{2}E}{\partial w_{1}\partial w_{2}}\\ \frac{\partial^{2}E}{\partial w_{2}\partial w_{1}} & \frac{\partial^{2}E}{\partial w_{2}^{2}} \end{array} & \;\cdots & \begin{array}{c} \frac{\partial^{2}E}{\partial w_{1}\partial w_{N}}\\ \frac{\partial^{2}E}{\partial w_{2}\partial w_{N}} \end{array}\\ \vdots & \ddots & \vdots \\ \begin{array}{cc} \frac{\partial^{2}E}{\partial w_{N}\partial w_{1}} & \frac{\partial^{2}E}{\partial w_{N}\partial w_{2}} \end{array} & \cdots & \frac{\partial^{2}E}{\partial w_{N}^{2}} \end{array}\right]$$

The updated values of the free parameters (Δ**w**^(k)^) can be calculated as follows [20]:

(8) $$\Delta \;w{\;}^{\left(k\right)}=-H^{-1}*G,$$

where **H**^−1^ is the inverse of the Hessian matrix of E(**w**), and **G** is the gradient of E(**w**).

The training subset, which has corresponding pairs of input variables (**x**_i_) and output variable (**d**_i_), is used to calculate the MSE function described by Equation (6). The optimisation process involves a continuous updating of the free parameters (weights and biases) from the initial values until reaching convergence between the approximated response (**y**_i_) and the true output (**d**_i_). The initial values of all biases are set to zero. The initial values of weights should be in the range of the activation function’s boundaries. The desirable values of the weights are calculated on the basis of the uniform distribution to have a mean of zero, and the variance is equal to the reciprocal of the number of connections at the hidden neuron. When **H** is a positive definite matrix, the convergence is simply performed by Newton’s method. However, this is not always the case, so a scalar parameter (μ) can be added to the diagonal elements of **H** to modify it. The value 1/(2μ) is called the learning rate (η), which has to be a very small value but must never reach zero. It is set to an initial value less than one. When the MSE is reduced, μ^(k+1)^ = μ^k^/2; otherwise, μ^(k+1)^ = 2μ^k^ if the MSE is increased. The convergence criterion is formulated on the basis of the local or global minimum of the error surface. If the updated values of **w**i achieve a local or global minimum, then the gradient of the updated free parameters (**G**) is zero [20,27]. Therefore, the most important advantage of using the FLNN is the ability to modify the **H** matrix and to find an optimisation solution even when the **H** matrix is not a positive definite matrix. Another important advantage of the FLNN is that it has the ability to deal with the nonlinearity of a function by processing computations in the hidden layer and various activation functions [20].

#### 2.2.2. Evaluation Criteria of the FLNN Model

The goal of the FLNN with supervised learning is to gain sufficient knowledge from the dataset in order to be able to predict the future response with an acceptable deviation from the exact solution for the specific criteria. The standard tool for achieving this goal is known as cross-validation [28]. In this context, the dataset was randomly divided into a training subset used to build the model (70%), a validation subset to examine the overfitting of the model (15%) and a testing subset used to generalise the model for future applications (15%) [20]. As stated, the most crucial criterion was the mean squared error (MSE); however, the correlation coefficient (R) between the model and the experimental response were also considered.

### 2.3. Controlling Factors and Description of the Data

Based on extensive research in the peer-reviewed literature [11,21,29,30,31,32,33,34,35,36,37,38,39,40,41,42,43], a dataset summarising 189 FA-based geopolymer concrete samples (N = 189) with various mix-designs (the input vector, **x**_i_) and the corresponding response (i.e., the compressive strength) (the output vector, **d**_i_) was collected. The input variables for geopolymer concrete were, however, not unified, and the authors did not follow a unified method. Through simple calculations, the datasets were converted into equivalent variables, which enabled a design method for FA geopolymer concrete that was similar to that of Portland cement concrete [44].

The following key points were applied to select the input variables on the basis of the chemical composition of the FA, the proportions of the ingredients and the curing conditions:

1. The chemical composition of the FA:

It has a significant effect on compressive strength. Five oxides can affect the chemical composition. The backbone oxides of the three-dimensional geopolymerisation network are SiO_2_, Al_2_O_3_ and Fe_2_O_3,_ as well as the alkaline oxides Na_2_O and CaO [23]. The relativity modulus was introduced to simplify the chemical composition in one parameter. However, RM combined the oxides (SiO_2_ and Na_2_O) from the activator [45]. According to [22], around 23% of FA oxides are not amorphous. Therefore, in this study, the following chemical composition index (CCI) was introduced to represent the effect of the chemical composition for the raw material only:

(9) $$\mathrm{CCI}=\frac{{\mathrm{Na}}_{2}O+\mathrm{CaO}}{{\mathrm{SiO}}_{2}+{\mathrm{Al}}_{2}O_{3}+{\mathrm{Fe}}_{2}O_{3}}$$

2. Ingredient proportions:
  ● FA ash content: The weight of the FA was taken in kg/m^3^ in the design-mix as the most solid component of the geopolymer solids (GS) [21,44].
  ● The effect and interaction of the aggregate are similar to those of Portland cement concrete [21,44]. Therefore, two parameters have been used. The first parameter was the coarse aggregate–total aggregate ratio (C_agg_/T_agg_ (kg/m^3^)), which reflects the effect of aggregate gradation. The second parameter was the geopolymer solids–total aggregate ratio (GS/T_agg_ (kg/m^3^)), which reflects the effect of the interaction between the total aggregate (filler material) and the geopolymer solids (matrix) in the composite.
  ● The superplasticiser was taken as a weight percentage of the raw material (FA) [21,44].
  ● Alkali activator and water: In the case of a two-part geopolymer, different terms have been used to define the variability of the alkali activator, such as the molarity of sodium hydroxide (M), the content of sodium hydroxide (SH), the sodium silicate module (Ms) and the sodium silicate–sodium hydroxide ratio (SS/SH). Both the sodium hydroxide solution and the sodium silicate solution were usually mixed 1 day prior to mixing the geopolymer concrete, producing the final sodium silicate (Na_2_SiO_3_) composition [21,44]. Interestingly, for one-part geopolymers, the alkali activator was solid sodium silicate (Na_2_SiO_3_) [46]. Therefore, Na_2_O% and SiO_2_/Na_2_O were directly calculated from the Ms. From a practical point of view, it is more feasible to convert the alkali activator solution’s variables (M, SH, Ms and SS/SH) to the equivalent of Na_2_O%, SiO_2_/Na_2_O ratio and water in the alkali solution rather than converting the solid alkali activator’s variables (Na_2_O%, SiO_2_/Na_2_O ratio). Calculations of the solid parts of the alkali activation solution were based on the chemical composition, molecular weight and weight of each component in kg/m^3^, as described by [21,44]:
    (10) $$\mathrm{NaOH}=\mathrm{SH}*{\%\;\mathrm{GS}}_{\mathrm{SH}},$$
    (11) $${\mathrm{Na}}_{2}O+{\;H}_{2}O\;\to 2\mathrm{NaOH},$$
    (12) $${\left({\mathrm{Na}}_{2}O\right)}_{\mathrm{SH}}=\frac{{\mathrm{Mw}}_{\left({\mathrm{Na}}_{2}O\right)}}{2{\;*\;\mathrm{Mw}}_{\left(\mathrm{NaOH}\right)}}*\mathrm{NaOH},$$
    (13) $${\mathrm{SiO}}_{2}=\;\mathrm{SS}*{\%\;\mathrm{GS}}_{{\mathrm{SiO}}_{2}},$$
    (14) $${\left({\mathrm{Na}}_{2}O\right)}_{\mathrm{SS}}=\;\mathrm{SS}*{\%\;\mathrm{GS}}_{{\mathrm{Na}}_{2}O},$$
    (15) $${\left({\mathrm{Na}}_{2}O\right)}_{T}={\left({\mathrm{Na}}_{2}O\right)}_{\mathrm{SH}}+{\left({\mathrm{Na}}_{2}O\right)}_{\mathrm{SS}},\;$$
    (16) $${\mathrm{Na}}_{2}O\;\%=\frac{{\left({\mathrm{Na}}_{2}O\right)}_{T}}{\mathrm{FA}},$$
    (17) $$\frac{{\mathrm{SiO}}_{2}}{{\mathrm{Na}}_{2}O}=\frac{{\mathrm{SiO}}_{2}}{{\left({\mathrm{Na}}_{2}O\right)}_{T}},$$
    (18) $$w\;=\mathrm{SH}*{\%\;w}_{\mathrm{SH}}+\mathrm{SS}*{\%\;w}_{\mathrm{SS}}+{\;w}_{\mathrm{ext}},$$
    (19) $$\mathrm{GS}=\;\mathrm{FA}+{\left({\mathrm{Na}}_{2}O\right)}_{T}+{\;\mathrm{SiO}}_{2},$$
    where NaOH is the weight of the hydroxide sodium solids in the sodium hydroxide solution, SH is the weight of the sodium hydroxide solution, % GS_SH_ is the weight percentage of the sodium hydroxide solids in the sodium hydroxide solution, Na_2_O is the sodium oxide, ${\left({\mathrm{Na}}_{2}O\right)}_{\mathrm{SH}}$ is the weight of the sodium oxide weight in the sodium hydroxide solution, ${\mathrm{Mw}}_{\left({\mathrm{Na}}_{2}O\right)}$ is the molecular weight of the sodium oxide, ${\mathrm{Mw}}_{\left(\mathrm{NaOH}\right)}$ is the molecular weight of the sodium hydroxide, ${\mathrm{SiO}}_{2}$ is the weight of the silicate solid in the sodium silicate solution, SS is the weight of the sodium silicate solution, ${\%\;\mathrm{GS}}_{{\mathrm{SiO}}_{2}\;}$ is the weight percentage of solid silicate in the sodium silicate solution, ${\left({\mathrm{Na}}_{2}O\right)}_{\mathrm{SS}}$ is the weight of sodium oxide in the sodium silicate solution, ${\%\;\mathrm{GS}}_{{\mathrm{Na}}_{2}O\;}$ is the weight percentage of sodium oxide in the sodium silicate solution, ${\left({\mathrm{Na}}_{2}O\right)}_{T}$ is the total weight of sodium oxide in the alkali activator, ${\mathrm{Na}}_{2}O\;\%$ is the percentage of the total sodium oxide in the alkali activator with respect to the weight of the fly ash, FA is the weight of the fly ash, w is the total weight of water in the FA-based geopolymer concrete, ${\%\;w}_{\mathrm{SH}}$ is the weight percentage of water in the sodium hydroxide solution, ${\%\;w}_{\mathrm{SS}}$ is the weight percentage of water in the sodium silicate solution, $w_{\mathrm{ext}}$ is the weight of the extra weight of water used to increase workability and GS is the total solids in the geopolymer binder.

Therefore, the alkali activator was represented in this research as the Na_2_O% and the SiO_2_–Na_2_O ratio to make the model applicable for both one-part and two-part FA geopolymer concrete. Another advantage of separating the alkali activator’s oxides from water is that water has a negative effect on compressive strength, while Na_2_O and SiO_2_ have positive effects on compressive strength [10,21]. Hence, the water in the FA geopolymer concrete mix was represented by the w/GS parameter.

3. Curing conditions:

FA geopolymer concrete is usually associated with heat curing at an early stage [10]. The curing condition parameters were based on the curing time (CT) in hours and the temperature (T) of curing in degrees Celsius.

**Figure 3:**
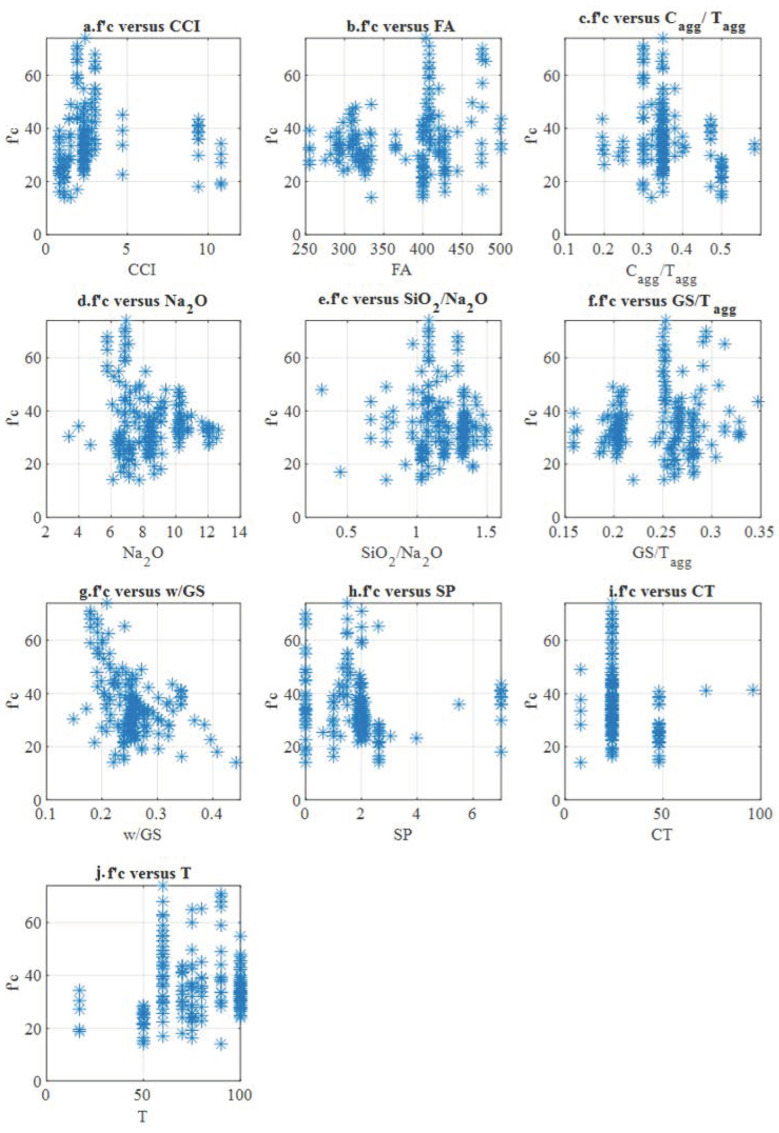
Compressive strength of the FA geopolymer concrete (f’c) versus selected input variables: (**a**) f’c versus CCI, (**b**) f’c versus FA, (**c**) f’c versus C_agg_/T_agg_, (**d**) f’c versus Na_2_O, (**e**) f’c versus SiO_2_/Na_2_O, (**f**) f’c versus GS/T_agg_, (**g**) f’c versus w/GS, (**h**) f’c versus SP, (**i**) f’c versus CT and (**j**) f’c versus T.

In the experimental determination of the ultimate compressive strength of the geopolymer concrete (as is the case for ordinary Portland cement concrete), the shape and size of the test specimens (cubes or cylinders), as well as the boundary conditions and speed of the test, influenced the results obtained [7]. Therefore, all the compressive strength values of cubes from the literature were converted into the corresponding cylindrical compressive strength values according to [47]. Thus, the cylindrical compressive strength at 28 days of age (f’c in MPa) represents the output variable in this study. The raw data with their equivalents can be seen in Table S1 in the Supplementary Materials of this article. The dataset with ten input variables and the output variable is shown in Table S2 of the Supplementary Materials. Statistical parameters such as the range, mean, median and standard deviation of the dataset were calculated and are presented in Table 1. Figure 3 illustrates the relationship between the output variable (the dependent variable, f’c) and each input variable (the independent variables). Almost none of the dependent variables showed a clear trend with the independent variables except for the w/GS variable, indicating that increasing the w/GS ratio decreases the compressive strength. This tendency may indicate that w/GS is the most crucial factor affecting FA-based geopolymer concrete. This finding agrees with the results of [21]. The ten input variables of the model are listed in Table 1.

## 3. Results and Discussion

### 3.1. Development and Optimisation of the FLNN Model in Matlab

**Figure 4:**
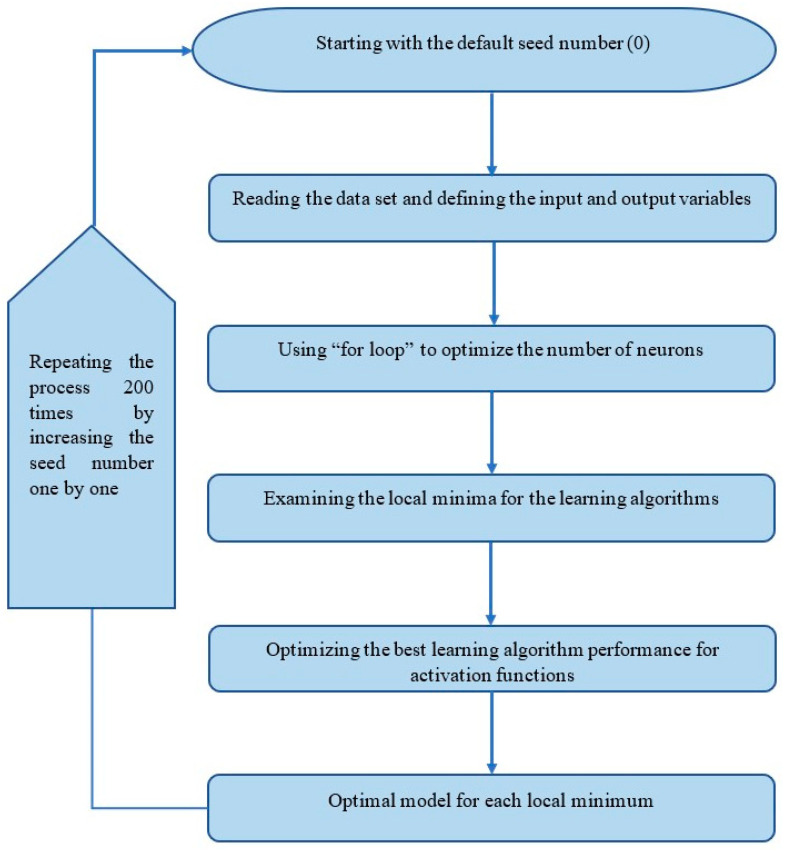
The development steps of the Matlab code optimisation process.

**Figure 5:**
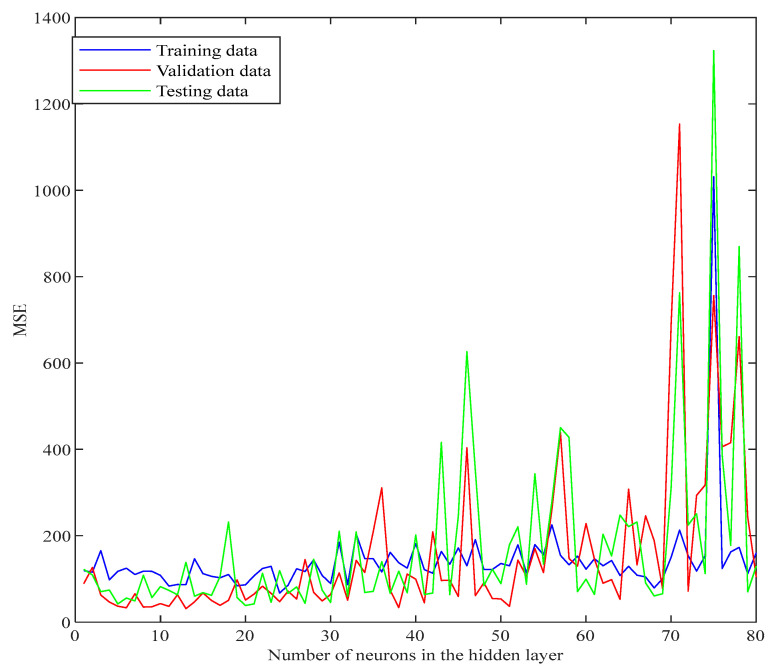
Optimisation of the number of neurons in the hidden layer based on cross-validation.

Through the definition of the FLNN, the optimisation process is governed by the number of hidden units (neurons), the choice of the learning algorithms and the choice of the activation function. In the optimisation process, 12 learning algorithms (*trainrp*, *trainlm*, *traincgp*, *traincgb*, *trainbfg*, *trainos*, *traincgf* and *traingda*) and eight activation functions (*tansig*, *radbas*, *poslin*, *elliotsig*, *elliot2sig*, *softmax*, *logsig* and *satlin*) already built into the Matlab software [24] were used for developing the code. The flowchart in Figure 4 shows the development steps of the Matlab code optimisation process, which consisted of the following:

1. Random seeds (S): In general, the error surface has many local minima; as a result, FLNN models would have a set of S that could yield better solutions than others [48]. Therefore, S should be assigned at the beginning of each trial of the code optimisation process.
2. Defining the input and output variables: After the dataset had been sorted into the required input and output variables, the dataset was stored in the target directory of the potential Matlab code in the form of a Microsoft Excel file. The relevant operations of matrices and arrays were used to define the input vector (**x**_i_) and output vector (**d**_i_) [20,49].
3. Optimising the number of neurons: The number of hidden neurons was changed from 1 to 80. For each FLNN with a specific number of hidden neurons, the MSE was calculated for the training, validation and testing subsets. This was carried out by generating a for-loop considering the number of hidden neurons, which varied from 1 to 80. The solution was evaluated through the cross-validation technique by plotting the MSE versus the number of hidden neurons [20,50]. The MSE converged at the local and global minima, which was a topic of interest to be investigated in the subsequent steps. Figure 5 shows the convergence and divergence of MSE versus the number of neurons in the hidden layer.
4. Determining the best performing learning algorithm: After choosing the number of hidden neurons corresponding to a specific local or global minimum, the performance of the FLNN was examined for all 12 learning algorithms through a cross-validation of the MSE. Only the training and validation data subsets were applied at this stage. Nevertheless, an interesting learning algorithm, the Bayesian Regularisation, is self-optimised and does not require a validation subset, but only needs to be assigned the training and testing subsets [16,24]. Therefore, the percentage of validation subset was set to 0 when Bayesian Regularisation was used in the following potential steps.
5. Optimisation for the choice of activation function: After selecting the best performing learning algorithm, the Matlab code ran this learning algorithm through all eight activation functions to identify the optimal choice of the activation function type.
6. Selecting the optimal model: By determining the number of the hidden neurons, the learning algorithm type and the activation function, the optimal model criteria such as MSE and R were recorded for each local or global minimum.

**Figure 6:**
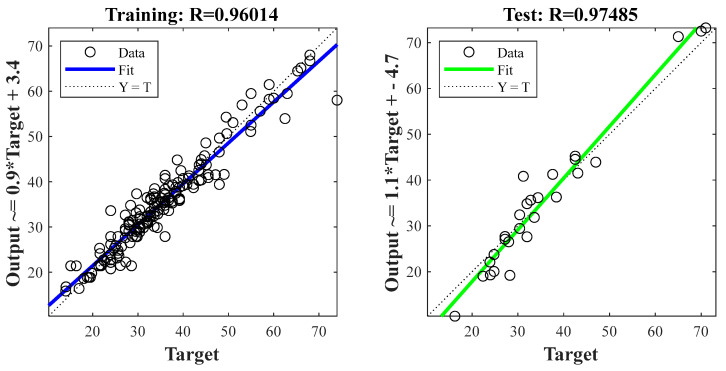
The correlation coefficient (R) of the training and testing subsets for the optimal model.

In the first step of the optimisation, the Matlab code was run 201 times by changing S (0–200). The local and global minima were investigated each time by selecting the corresponding hidden neurons. After that, the learning algorithm and activation function choices were examined to determine the optimal model. The best performance was achieved when the default seed number (S = 0) was applied. The results of S = 0 are presented in Table 2. The selection criteria of the optimal model were the number of hidden neurons (H), the learning algorithm (LA) type and the activation function (AF) type, based on the MSE function and the R between the model output and the actual output. The analysis showed that the best performance was found for Model No. 4. The architecture of Model No. 4 consists of seven hidden neurons, a *trainbr* learning algorithm and a *tansing* activation function [16,24]. As mentioned earlier, the *trainbr* algorithm does not require validation subsets. Therefore, the validation subset ratio was set to zero, and the performance parameters (MSE and R) were evaluated for only the training and testing subsets. The MSE values were 10.4 and 15.0 for the training and testing subsets, respectively. The root mean squared error (RMSE) values were calculated by taking the root of the MSE values, thus giving ±3.22 MPa and ±3.87 MPa, respectively. As shown in Figure 6, the R values were 96.0% and 97.5% for the training and testing subsets, respectively.

**Figure 7:**
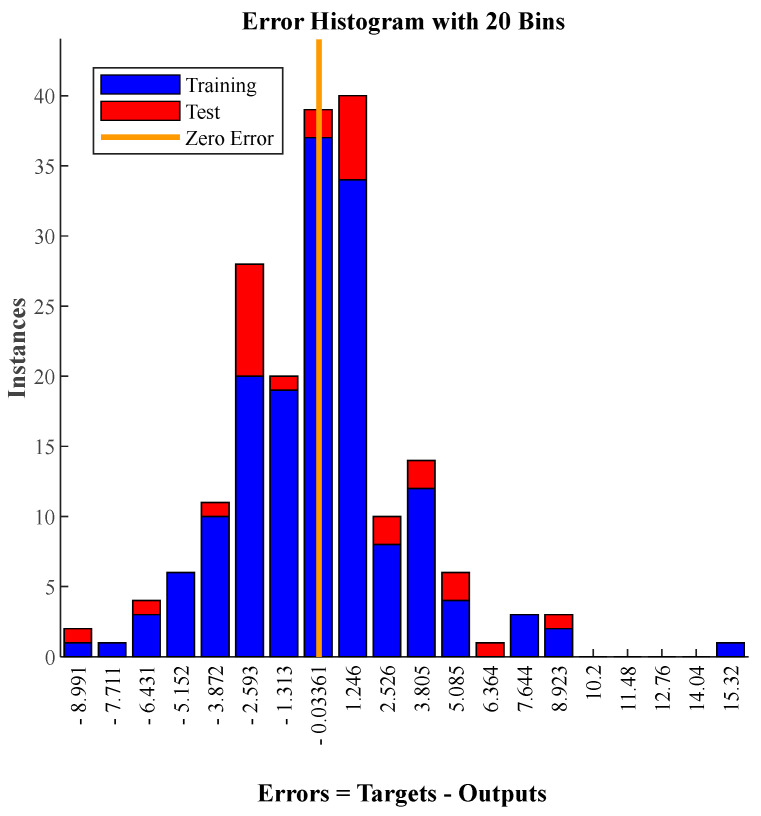
Error histogram of the optimal model.

Since the RMSE value was ±3.87 MPa for the testing data (data that were unseen in the training process), the optimal model could be generalised for future data with an average error of ±3.87 MPa. Figure 7 demonstrates that the error of the optimal model is normally distributed, and most of the data have an error value equal to or less than the RMSE (±3.87 MPa). Since the RMSE follows a normal distribution, the probability can be calculated from the Gaussian probability tables [25]. By applying the z probability table, for z = ±3.87, the corresponding probability value is 99.995%. This means that the probability of the optimal model would be 99.995% in the [−3.87, 3.87] interval for future data. Therefore, the performance of the optimal model is excellent compared with the results of other machine learning methods in the peer-reviewed literature.

It has been repeatedly reported that the w/GS ratio is the most significant factor controlling the compressive strength [10,21]. However, it only had a small range (0.17–0.22) in the mix-design method described by Lloyd and Rangan [10]. The optimal model in this study was utilised to investigate the influence of w/GS on the compressive strength. The optimal model was run after eliminating w/GS. The MSE and R values were significantly affected, reaching 24.8 MPa^2^ and 46.0 MPa^2^, and 90.2% and 86.7% for the training and testing subsets, respectively, indicating high error signals. Therefore, MSE increased by 206% for the testing subset (unseen, future data). This finding agrees with Hardjito and Rangan [21], and Lloyd and Rangan [10], who stated that the most significant factor controlling the compressive strength is w/GS. Therefore, the optimal model is more flexible, since the w/GS range has been increased to 0.15–0.44, corresponding to a 580% change, which is much wider than the one described by Lloyd and Rangan [10].

Researchers have not reached common ground about the chemical composition of the raw material used in geopolymer concrete, although it indisputably has a considerable effect on compressive strength [22,23]. This study introduced CCI to represent the chemical composition of the raw material in terms of five major oxides. The optimal model was run after eliminating the CCI from the input variable matrix. The results showed that the MSE, RMSE and R values changed to 20.8 and 19.7 MPa^2^, 4.56 and 4.44 MPa and 92.3% and 94.1% for the training and testing subsets, respectively, indicating a considerable increase in the error signals. Thus, MSE increased by 31.3% for the testing subset (unseen, future data). Toufigh and Jafari [23] have also considered the effect of the chemical composition by using five major oxides. However, the proposed optimal model of this research surpassed the results of the MR model of [23], where the RMSE and R values were 4.83 and 5.96 MPa and 89.0% and 82.6% for the training and testing subsets, respectively. The proposed optimal model of this study has reductions in the RMSE values of 33.3% and 35.1%, while the R values increased by 7.9% and 18.1% for the training and testing subsets, respectively. The difference in the two outcomes is believed to be due to the consideration of the nonlinear relationship of the proposed model in this study. This assumption is supported by the findings of Ahmed et al. [14] that a nonlinear relationship is the most suitable representation of the compressive strength of FA geopolymer concrete.

### 3.2. Generation of the FA Geopolymer Concrete Mix-Design Chart

Practicing civil engineers do not have the required computers, software and knowledge in programming. Thus, a reliable and feasible design chart with a wide range of input variables could strongly support the design procedure of geopolymers’ structural elements under a compression load. Aiming this, a design chart was generated by the optimal model developed by the Matlab code of the FLNN, which presents the relationship between the compressive strength (output variable) and the most significant input influencing variable w/GS ratio for a specific level of all other input variables.

**Figure 8:**
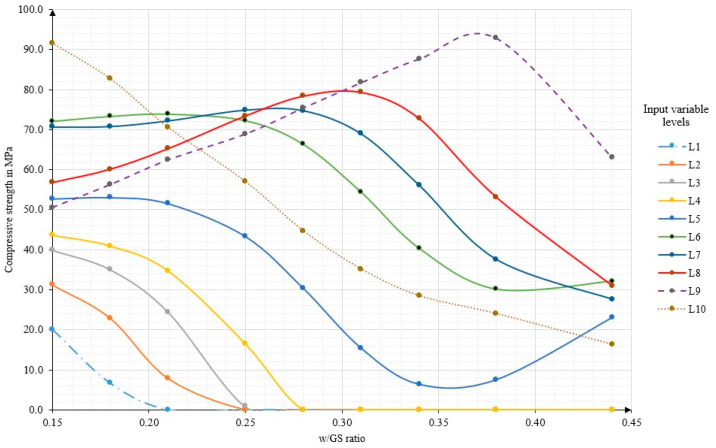
Compressive strength (f’c) vs. the water–geopolymer solid (w/GS) ratio for the ten levels of the input variables (L1–L10).

Starting from the minimum values of each input variable and ending with the maximum values, there were nine levels (L1–L9) of each input variable in equal steps. In addition, the 10th level (L10) was established for the mean values of each input variable. Table 3 shows these ten levels and their corresponding variables. Therefore 10 (10 × 10) matrices were established and run by the Matlab code to calculate the predicted output value based on the optimal model. These results are plotted against the w/GS variable in Figure 8. The compressive strength vs. w/GS ratio curves can be classified into five types, as shown in Figure 8. The first family consists of the L1, L2, L3 and L4 curves. The variation in the compressive strength starts from an acceptable value at the lowest w/GS ratio. By increasing the w/GS ratio, the compressive strength tends to decrease according to a nonlinear relationship and reaches zero after the third, fourth, fourth and fifth levels of the w/GS ratio for L1, L2, L3 and L4, respectively. Second, for the curve at the fifth level (L5), the compressive strength shows a stable variation at the first three levels of the w/GS ratio. After four levels of the w/GS ratio, the compressive strength undergoes a sharp decrease until it reaches the seventh level of the w/GS ratio and starts increasing again. The third family consists of the L6 and L7 curves. The compressive strength starts and continues at a steady rate until reaching Level 5 of the w/GS ratio, then starts decreasing smoothly. The fourth family consists of L8 and L9. The compressive strength starts at considerably lower values than for L6 and L7 at the lowest w/GS ratio. The reason for this is probably the high alkalinity of these levels (L8 and L9), which requires a greater amount of water. It has been reported that H_2_O and Na_2_O are directly connected in the geopolymerisation process [44]. By increasing the w/GS ratio, the compressive strength gradually increases until reaching the optimum value at the sixth and eighth levels of the w/GS ratio for L8 and L9, respectively. The fifth family, L10, represents the input variables at mean values. The compressive strength starts at a considerably high value (91.6 MPa), corresponding to a w/GS ratio of 0.15. The compressive strength decreases almost linearly with an increase in the w/GS ratio, reaching the minimum (16.3 MPa) at a w/GS ratio of 0.44. The reason behind the change in each curve in the design chart is obviously due to the difference in the microstructure of the geopolymer matrix. The microstructure of the geopolymer matrix is substantially dependent on the molar ratio of the synthesis oxides [6,44]. By altering the molar ratio of the synthesis oxides, the silico-aluminate can have several possible three-dimensional structures (e.g., polysilicate, polysiloxonate and polysialate) in a geopolymerisation process. These three-dimensional structures differ in their geometry and their bond strength. Moreover, the distribution of the alkali cations such as (Na^+^) required to balance the negative sign of tetracoordinated Al(4) atom [6] also plays a significant role in the stability of the geopolymer matrix. Therefore, the different mix-designs of the geopolymer matrix induce different relationships [6].

The design procedure is as follows:

1. The specified target compressive strength of the FA-based geopolymer concrete (obtained from the structural design) is selected on the *y*-axis and horizontally projected onto the individual curves of the input variable levels (L1–L10) depicted in Figure 8. It is apparent that for the same target compressive strength, several possible mix-designs exist (intersection points), each with its own corresponding w/GS ratio (on the *x*-axis).
2. The designers have the flexibility to choose one of the mix-design combinations (based either on the preferred w/GS ratio or any other requirements/restrictions of the mix-design parameters). The particular values of each mix-design parameter (input variables) are then read out from Table 3 for the selected L level. Since the compressive strength and w/GS are not constant, this characteristic gives more flexibility to different levels for the other input variables for the same target compressive strength.

The design chart developed here is a powerful tool for structural engineers, supporting them in the selection and calculation of the mix-design parameters of FA-based geopolymer concretes with the composites’ targeted compressive strength.

Lloyd and Rangan [10] generated a mix-design chart for predicting the compressive strength of FA-based geopolymers based on a w/GS ranging from 0.17 to 0.22. Their mix-design chart consisted of only three levels and changed only one other controlling factor, namely curing temperature, which had a range of 30–90 °C. All the curves had a similar trend to L10, where the controlling factors had the mean values in the present study. The reason for this similarity to only the L10 curve could be because there was a large number of variables that had been fixed at values equal to or near the mean values. Moreover, the aforementioned study did not consider the chemical composition of the raw material. It can be inferred that the relationship between the compressive strength of FA-based geopolymer concrete and the w/GS ratio is not constant and is influenced by the levels of the other input variables. Interestingly, some parts in the design chart showed a constant compressive strength despite a change in all the input variables, which is called robust design. The design chart shows a robust mix-design for the L6 and L7 curves when the w/GS ratio equals or is less than 0.28 because of the wide range of the input variable levels that allows one to discover this robust mix-design region where deviation in the input variables does not affect the compressive strength negatively. After increasing the w/GS ratio to more than 0.28, the compressive strength starts to decrease smoothly.

## 4. Techno-Economic Challenges and Future Recommendations

Geopolymer production can be profitable only when the dosage of the alkali activator is controlled [51]. An increase in the geopolymer concrete’s compressive strength needs an increase in the dosage of the alkali activators in the matrix [21], which, in turn, significantly increases the cost of the composite. The design chart proposed here, which has been based on the optimal model, has a narrow uncertainty margin, thus providing a reliable tool for predicting the target strength in the vicinity of the true compressive strength. Hence, it efficiently controls the geopolymers’ production cost by avoiding unnecessary access costs due to the higher target strength of the mix-design.

Two-part geopolymer production is considered very challenging in many practical applications due to the difficulties of preparing the alkali activator solution and the process of mixing this with the solid precursor, which requires stringent preventative safety requirements [46]. On the other hand, one-part geopolymers can be manufactured also by not so well-trained workers in a very similar way to Portland cement concrete. Therefore, moving from two-part geopolymer formulations to one-part during production is a pivotal step in making geopolymer production able to compete with traditional Portland cement.

## 5. Conclusions

In this research, the compressive strength of FA-based geopolymer concrete was predicted by an optimisation process via Matlab code using the FLNN technique. The training and testing datasets were separated into ten input variables (mix proportion, curing conditions and chemical composition of the raw material) and into the output variable (compressive strength). The Matlab code was run 201 times. Each time, the hidden neuron number at the local and global minima were checked to determine the learning algorithm type and to choose the activation function, respectively.

As a result, the following main conclusions can be stated:

- The FLNN network proved its superior behaviour over other machine learning techniques by obtaining better performance criteria for predicting the compressive strength of FA-based geopolymer concrete. The proposed optimal model achieved reductions in the RMSE values of 33.3% and 35.1%, while the R values increased by 7.9% and 18.1% for the training and testing subsets, respectively, compared with the best performing model in the literature [23].
- The proposed model is capable of predicting the compressive strength of FA geopolymer concrete at 28 days of age within a small range of deviation from the actual values (±3.87). It has a high correlation with the actual values and a relatively small error on average, with a very high R value (97.5%) for the unseen data.
- The optimal model is applicable to both one- and two-part geopolymer concretes. Moreover, it considers a wide range of mix-design parameters, such as the chemical composition index, the FA content, the coarse aggregate–total aggregate ratio, the Na_2_O percentage, the SiO_2_–Na_2_O ratio in the activator, the geopolymer solids–total aggregate ratio, the water–geopolymer solid ratio, the percentage of superplasticiser and the curing time and temperature.

Additionally, based on the proposed optimal model, a powerful design chart, which is applicable to both one- and two-part geopolymer concretes, has been generated that considers a wide range of mix-design combinations for the targeted compressive strength of the FA geopolymer concrete. The design chart provides a strong support for other experts in the design of the mixtures, simplifying and facilitating the design process itself in a cost- and time-efficient way, while leaving the designer high flexibility.

## Figures and Tables

**Table 1:** Description and summary statistics of the input and output variables.

No.	Variable (Unit)	Description	Type of Variable	Range	Mean	Median	St. Dev. ^1^
1	CCI (%)	chemical composition index	input	0.80–10.80	2.68	2.31	2.25
2	FA (kg/m^3^)	fly ash content	input	255.0–500.0	375.98	400	60.38
3	C_agg_/T_agg_ (kg/m^3^)	coarse aggregate–total aggregate ratio	input	0.20–0.58	0.36	0.35	0.07
4	Na_2_O (% wt)	sodium oxide–fly ash ratio	input	3.39–12.64	8.37	8.32	1.79
5	SiO_2_/Na_2_O (kg/m^3^)	silicon dioxide–sodium oxide ratio in the activator	input	0.32–1.50	1.18	1.19	0.19
6	GS/T_agg_ (kg/m^3^)	geopolymer solids–total-aggregate ratio	input	0.16–0.35	0.25	0.25	0.04
7	w/GS (kg/m^3^)	water–geopolymer solid ratio	input	0.15–0.44	0.25	0.25	0.04
8	SP (%)	superplasticiser as a percentage of the fly ash weight	input	0.0–7.00	1.89	1.94	1.48
9	CT (hours)	heat curing time	input	8.00–96.00	27.89	24.00	10.97
10	T (°C)	curing temperature	input	17.00–100.00	78.25	75.00	20.47
11	f’c (MPa)	compressive strength	output	14.05–74.0	35.29	33.60	11.85

^1^ Standard deviation.

**Table 2:** Selecting the optimal model based on the Matlab code results.

No.	H ^1^	LA ^2^	AF ^3^	MSE (MPa^2^)			R (%)		
				Training	Valid. ^4^	Test	Training	Valid. ^4^	Test
1	4	*trainbr*	*elliot2sig*	25.6	NA	48.9	90.5	NA	78.2
2	5	*trainbr*	*logsig*	12.7	NA	165	95.0	NA	54.2
3	6	*trainbr*	*logsig*	15.0	NA	30.5	95.0	NA	91.1
4	7	*trainbr*	*tansig*	10.4	NA	15.0	96.0	NA	97.5
5	9	*trainbr*	*tansig*	7.3	NA	43.2	98.0	NA	75.3
6	11	*trainbr*	*tansig*	3.1	NA	131.5	98.9	NA	63.4
7	12	*trainbr*	*logsig*	5.6	NA	65.8	98.0	NA	76.2
8	15	*trainbr*	*logsig*	5.5	NA	33.7	98.0	NA	86.7
9	17	*trainbr*	*logsig*	5.4	NA	129.9	98.1	NA	75.3
10	19	*trainbr*	*elliotsig*	6.6	NA	51.2	97.7	NA	83.3
11	20	*trainbr*	*elliotsig*	8.0	NA	11.7	97.4	NA	89.6
12	24	*trainbr*	*logsig*	6.4	NA	32.6	97.6	NA	90.0
13	30	*trainbr*	*radbas*	8.2	NA	103.6	97.2	NA	64.9
14	32	*trainlm*	*radbas*	72.9	38.8	96.5	80.1	83.1	81.6
14	39	*trainbr*	*logsig*	5.0	NA	89.1	97.9	NA	81.2
16	41	*trainbr*	*elliot2sig*	8.4	NA	42.3	97.0	NA	82.7
17	48	*trainbr*	*tansig*	4.4	NA	43.4	98.4	NA	81.2
18	53	*trainlm*	*satlin*	10.6	81.5	166.0	96.5	75.4	66.0
19	69	*trainbr*	*radbas*	21.4	NA	65.0	92.2	NA	74.7
20	80	*trainbr*	*elliotsig*	5.5	NA	64.4	97.9	NA	81.1

^1^ Number of hidden neurons; ^2^ learning algorithm; ^3^ activation function; ^4^ validation; underlined the best performance model.

**Table 3:** The levels of the input variables (L1–L10) for generating the design chart.

Input Variables	Level of Input Variables									
	L1	L2	L3	L4	L5	L6	L7	L8	L9	L10
CCI (%)	0.80	1.91	3.02	4.13	5.24	6.36	7.47	8.58	10.80	2.68
FA (kg/m^3^)	255	282	309	337	364	391	418	446	500	376
C_agg_/T_agg_ (kg/m^3^)	0.20	0.24	0.28	0.33	0.37	0.41	0.45	0.50	0.58	0.36
Na_2_O (%wt)	3.39	4.42	5.45	6.47	7.50	8.53	9.56	10.58	12.64	8.37
SiO_2_/Na_2_O (kg/m^3^)	0.32	0.45	0.58	0.71	0.84	0.98	1.11	1.24	1.50	1.18
GS/T_agg_ (kg/m^3^)	0.16	0.18	0.20	0.22	0.24	0.27	0.29	0.31	0.35	0.25
w/GS (kg/m^3)^	0.15	0.18	0.21	0.25	0.28	0.31	0.34	0.38	0.44	0.25
SP (%)	0.00	0.78	1.56	2.33	3.11	3.89	4.67	5.44	7.00	1.89
CT (hours)	8.00	17.78	27.56	37.33	47.11	56.89	66.67	76.44	96.00	27.89
T (°C)	17	26	35	45	54	63	72	82	100	78

## Data Availability

Data from this study can be made available upon request.

## Supplementary Materials

The following supporting information can be downloaded at https://www.mdpi.com/article/10.3390/polym14071423/s1. Table S1: The raw data with their equivalents. Table S2: The dataset of the 10 input variables and the output variable.
